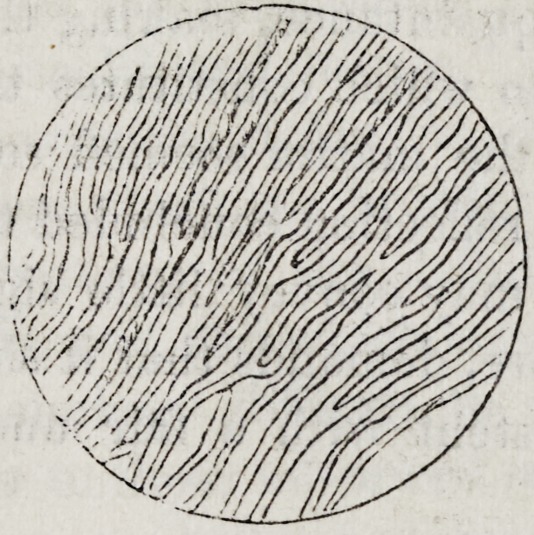# Bone Fibrous

**Published:** 1871-06

**Authors:** George B. Harriman

**Affiliations:** Prof. of Microscopic Anatomy, Boston Dental College.


### AMERICAN JOURNAL OF DENTAL SCIENCE.

Vol. V. THIRD SERIES—JUNE, 1871. No. 2.

### ARTICLE I.

### *Bone Fibrous.*

By George B. Harriman, M.D., D.D.S.

Prof. of Microscopic Anatomy, Boston Dental College.

The law of progress demands the multiplication of books on science and art. New developments are constantly occurring. Old theories and practices, the seeming embodiments of the wisdom of the past, and universally accepted as truth, like old fashioned garments are discarded, and superseded by more modern ideas, and appliances.

The medical, surgical and dental professions, have felt the electric impetus, and bound forward in the race with the vigor of youth and immortal truth.

The patient slowly consuming with fever, was but a few years ago denied water to allay his burning thirst, and fresh air was rigidly excluded from his apartment.

Now plenty of water and thorough ventilation is allowed with the most beneficial results. New theories have obtained with regard to the structure of the human body, mere notions, unsubstantiated by facts, were advocated in regard to human bone. Sections of bone were ground down thin, the vessels gradually becoming invisible, and the *cavities* containing these vessels, were termed *medullary canals.* The error of this theory is evident from the fact that these *narrow spaces contained no marrow.*

Bone corpuscles, and their processes, bone canals (CANALICULI OSSI), were common terms. At another period it was supposed they contained a deposit of calcareous matter ; this supposition was confirmed by its presence when viewed under the microscope, thus giving rise to another term, CANALICULI CHALICOPHORO. It was however found that no lime was contained in what was termed canals, but the lime was diffused in the bone. When, however, it was discovered that the calcarious salts had been erroneously located, resort was had to the theory that no lime existed in bone, and that it contained nothing but cavities and canals permeated by a fluid. These cavities were called fissures, and by some writers *bone fissures.* The latest, and now generally received theory, is that bone contains "structures provided with special walls and boundaries of their own, which separate them from the intermediate substance." These are termed *real corpuscles*, *atoms*, *bodies.* It is asserted that by the use of hydrochloric acid we are enabled by dissolving the basis-substance, to disengage the corpuscles from the intermediate substance, and thus demonstrate that they are really independent structures. Within these corpuscles or bodies, a nucleus may be distinguished, in which are cellular elements of stellate form. Bone, therefore, it is claimed, " exhibits in its composition a tissue containing in an apparently altogether homogeneous basis-substance peculiar stellate bone cells, distributed in a very regular manner."

This is Prof. Virchow's theory, and was first discovered by him more than fifteen years ago—an authority highly esteemed by the medical and surgical practitioner. Sir Lionel S. Beale, in his recent investigations, confirms Virchow and calls it " germinal matter."

While these eminent medical men have my esteem, and most profound respect for their *invaluable contributions to science*, yet repeated experiments, and careful analysis, have led me to entirely different conclusions. My theory is that the component parts of bone are *fibres* and *calcareous salts.* I have repeatedly viewed sections of bone, cementum and dentine, as thin as the three thousandth of an inch, which can be turned off in a lathe.

The above cut represents a thin shaving of bone, with the hard part (calcareous salts) dissolved with Prof. Hosford's acid phosphate, and is magnified two hundred diameters; and I have not found canals, nor tubes, nor fluids, nor cells, nor hollow spaces containing air, but FIBRES AND LIME SALTS.

The dentine of the tooth is composed of fibers and calcareous salts it is the same with the cementum; the fibres conveying nutrition to the bone, dentine, and cementum. Between bone, cementum, dentine, and plant, and tree, there is analogy. In the tree there are no canals or tubes, but fibers, and the fibers convey and diffuse nourishment for the trunk, branches, and roots of the tree.

---

## Figures and Tables

**Figure f1:**